# E2A suppresses invasion and migration by targeting YAP in colorectal cancer cells

**DOI:** 10.1186/1479-5876-11-317

**Published:** 2013-12-26

**Authors:** Hongchao Zhao, Ao Huang, Pu Li, Yingjun Quan, Bo Feng, Xuehua Chen, Zhihai Mao, Zhenggang Zhu, Minhua Zheng

**Affiliations:** 1 Department of Surgery, Shanghai Key Laboratory of Gastric Neoplasms, Shanghai Institute of Digestive Surgery, Ruijin Hospital, School of Medicine, Shanghai Jiao Tong University, 197 Rui Jin Er Rd, Shanghai 200025, People’s Republic of China; 2 Shanghai Minimally Invasive Surgery Center, Shanghai, China

**Keywords:** Metastasis, E2A, Colorectal cancer, YAP, EMT

## Abstract

**Background:**

E2A gene, which encodes two basic helix–loop–helix (bHLH) transcription factors E12 and E47, has been identified as regulator of B lymphoid hematopoiesis and suppressor of lymphoma. E47 protein was found to decrease E-cadherin expression and induce epithelial-mesenchymal transition (EMT). However, the role of E2A in colorectal cancer (CRC) metastasis is still elusive.

**Methods:**

qRT-PCR and semi-qRT-PCR were performed to determine mRNA level of E2A in CRC specimens and colorectal cancer cells. RNAi was employed to downregulate E2A expression and subsequent protein level change was evaluated by immunoblot. Cell invasion and migration capacity were detected by transwell assay using cell culture inserts with or without basement membrane matrix, respectively.

**Results:**

E2A expression was decreased in metastatic CRC tissues. Invasion and migration assays showed downregulation of E2A increased metastatic capacity of CRC cells while forced expression of E12 or E47 could offset this effect. Both E12 and E47 suppressed EMT induced by E2A downregulation. Moreover, Yes-Associated Protein (YAP) was a downstream target of E2A and suppression of YAP inhibited the pro-migration/invasion of E2A deficiency.

**Conclusion:**

Our results suggest that E2A suppresses CRC cell metastasis, at least partially if not all, by inhibiting YAP expression.

## Background

Colorectal cancer (CRC) is one of the most prevalent malignancies worldwide, ranking the third of all cancer-related deaths, and distant metastasis is the major cause of deaths for CRC patients [1]. These secondary tumors arise as the result of a multi-step process which begins when cancer cells within primary tumors break away from the microenvironment and invade through the basement membrane [2]. Although many metastasis-related genes have been identified in CRC [3], the complicated molecular mechanism of CRC development and progression is not yet fully understood.

The E2A gene encodes two basic helix–loop–helix (bHLH) transcription factors, E12 and E47, by means of variant splicing [4]. The E2A proteins (E12/E47) belong to the class I bHLH family and regulate expression of target genes by binding DNA with tissue-specific Class II HLH proteins, either as homodimers or as heterodimers [5-7]. Previous studies have shown that E12/E47 proteins are distributed widely in most adult tissues although at different expression levels [8,9]. Both proteins are indispensable for some normal tissue development while deficiency or aberrant expression of E2A could lead to tumorigenesis. For instance, E12/E47 proteins are required for B-cell differentiation and Ig gene rearrangements [10]; two aberrant fusion proteins, E2A-HLF and E2A-PBX1, lead to pro-B cell acute lymphoblastic leukemia (ALL) and pre-B cell ALL [11]. In addition, targeted disruption of the E2A gene leads to thymic lymphomas, suggesting that E2A gene products can act as tumor suppressors [12,13]. Specifically, the expression of epithelial maker E-cadherin was repressed upon E12/E47 binding to the E-pal element, a palindromic sequence between -75 and -86 in the E-cadherin promoter [14].

In colorectal cancer, ectopic expression of the E47 efficiently sequestered endogenous Id proteins from Id-bHLH heterodimers, leading to growth arrest of the cells [15]. However, it still remains unknown whether E12/E47 proteins affect CRC cells metastasis or not. In the present study, we demonstrated that E2A down-regulation was required for the development of metastasis while ectopic expression of E2A in CRC cells could suppress invasion and migration *in vitro*. Furthermore, we identified, and validated YAP (Yes-Associated Protein) as a novel downstream target of E2A.

## Methods

### Cell culture

Human colorectal cancer cell lines, SW480 and Caco-2, were purchased from American Type Culture Collection (ATCC). SW480 was cultured in Leibovitz’ L-15 Medium (Corning Cellgro®, Manassas, VA, USA) with 10% fetal bovine serum (FBS) (Invitrogen, Carlsbad, CA, USA), and Caco-2 in Minimum Essential Medium (Corning Cellgro®) with 20% FBS. Cells were maintained at 37°C/5% CO_2_ in a humidified incubator.

### Clinical specimens

The clinical research protocol was approved by the Ethical Committee of Ruijin Hospital, Shanghai Jiao Tong University School of Medicine. Seventy-five surgical specimens of primary CRC tumors were obtained from Shanghai Minimal Invasive Surgery Center (2012–2013) with written informed consents given by all patients prior to surgery. Inclusion criteria were: without neoadjuvant chemoradiotherapy; resectable colorectal cancers; without evidences of primary tumors of other organs; able to be interviewed during the follow-up. Fresh tumor tissues were harvested immediately after samples’ dissection, snap-frozen in liquid nitrogen, and preserved at -80°C. The pathologic classification/staging of tumors was performed in accordance to the Cancer Staging Manual from the International Union Against Cancer (7^th^ edition, 2009). Tumor samples were classified into two groups: tumors with metastatic sites and tumors without metastases, based on the status of lymph node and/or distant metastasis.

### Protein extraction and immunoblot

RIPA buffer (Solarbio, Beijing, China) containing protease inhibitor cocktail (Roche Applied Science, Basel, Switzerland) was used to extract total tissue and cell proteins, according to manufacturer’s instruction. Immunoblot was done according to the standard protocol, with primary antibodies against E2A (Santa Cruz, Dallas, Texas, USA), vimentin (Santa Cruz), E-cadherin (Cell Signaling Technol., Danvers, MA, USA), β-catenin (Cell Signaling Technol.), YAP (Cell Signaling Technol.) and MMP-9 (Cell Signaling Technol.). Goat anti-mouse or goat anti-rabbit IgG conjugated to horseradish peroxidase (HRP, Pierce, Rockford, IL, USA) was used as the secondary antibody. Chemiluminescent signals were visualized using SuperSignal West Pico Chemiluminescent Substrate (Pierce) and the signal intensity was analyzed using the Image Lab™ Software Version 4.0.1 (BIO-RAD, Hercules, CA, USA). The experiments were performed in triplicate with GAPDH (Kangchen, Shanghai, China) as endogenous control.

### Immunofluorescence

Cells growing on slides (Millipore, Billerica, MA, USA) were fixed with 4% paraformaldehyde (PFA, Sigma-Aldrich, St.Louis, USA) and permeabilized for 10 min using 0.1% TritonX-100/phosphate buffered saline (PBS). Non-specific antigens were then blocked with 3% BSA in PBS-T (0.2% Tween 20) for 1 h at room temperature. After washing, cells were incubated with primary antibodies against vimentin (Santa Cruz), E-cadherin (Cell Signaling Technol.), β-catenin (Cell Signaling Technol.) in 4°C overnight, followed by incubation with Alex Fluor® 555 anti-Rabbit IgG (Cell Signaling Technol.) for 2 hours at room temperature. Images were examined and captured using an Olympus Fluoview Confocal Microscope. Rabbit mAb IgG XP isotype (Cell Signaling Technol.) was used as negative control.

### Invasion and migration assay

Cell invasion and migration assays were performed using cell culture inserts (Millipore) coated with or without basement membrane matrix (BD Bioscience, Bedford, MA, USA), respectively. Assays were performed as previously described [16,17]. In brief, approximate 1 × 10^5^ cells resuspended in 200 μl non-serum culture medium were placed triplicatedly in upper chamber of insert and medium with 10% FBS was used as chemo-attractant in lower chamber; inserts were incubated at 37°C for 48 hours in a 5% CO_2_ humidified incubator. Cells attached on the inner side of the chamber were then cleared softly with cotton swab and cells outside the insert were stained in 1% crystal violet for 30 minutes. Cells in five random fields were counted under microscope and the relative invasion and migration capacity were interpreted as the average number of cells ± SD per field.

### RNA extraction and RT-PCR

Total RNAs from cell lines and tissues were extracted using Trizol reagent (Invitrogen) according to the manufacturer’s instruction. Reverse transcription of RNAs was performed using GoScript™ Reverse Transcriptase System (Promega, Madison, WI, USA) as per protocol. The mRNA level of YAP (forward: 5′- TAGCCC TGCGT AGCCA GTTA -3′; reverse: 5′- TCATGC TTAGT CCACTGTCTGT -3′) in cell lines was assessed by semi-qRT-PCR using Taq PCR MasterMix (Tiangen, Beijing, China). GAPDH (forward: 5′-GGAGC GAGAT CCCTC CAAAAT-3′; reverse: 5′- GGCTG TTGTC ATACT TCTCA TGG-3′) was used as an internal loading control. The expression of E2A (forward: 5′- CCACTT CACTG AGTCGC ACAG -3′; reverse: 5′- GTCTCT CCCGAA GGAGG CATA -3′) and YAP in tumor tissues were detected by real-time qRT-PCR using SYBR Green PCR Master Mix (Invitrogen) on the Applied Biosystems 7900HT sequence detection system with GAPDH as endogenous control.

### Transient transfection

Short hairpin RNA (shRNA) against human YAP and shRNA negative control (shNC) were bought from GenePharma (Shanghai, China). Plasmids pEZ-M29-E12 and pEZ-M29-E47, encoding fusion protein of eGFP-E12 or eGFP-E47 respectively, were purchased from Genecopoeia (Rockville, MD, USA). Cells were seeded in 6-well culture plates one day before transient transfection, which was performed with lipofectamine 2000 (Invitrogen), according to the instruction of manufacturer. Forty-eight hours after transfection, cells were harvested and the protein levels of the targeted genes were assessed by immunoblot, with GAPDH as loading control.

### Lentiviral transfection for stable expression clones

Plasmids pL/shRNA/F-shR with shE2A or shNC (negative control), namely LV-shE2A and LV-shNC, were purchased from Novobio (Shanghai, China). Lentivirus transfection was performed according to the manufacturer’s instruction to establish shE2A-expressing stable clones in SW480 cells (SW480/shE2A). The control clone (SW480/shNC) was constructed similarly. E2A protein expressions of abovementioned clones were examined by immunoblot using GAPDH as loading control.

### Statistical analysis

Two-tailed Student’s *t-*test, Spearman’s correlation or one-way ANOVA were used for statistical analysis when appropriate. All statistical analyses were performed using the SPSS 16.0 (SPSS Inc., Chicago, IL, USA). A two-tailed value of p < 0.05 was considered statistically significant.

## Results

### Expression of E2A was decreased in metastatic CRCs

**Figure 1:**
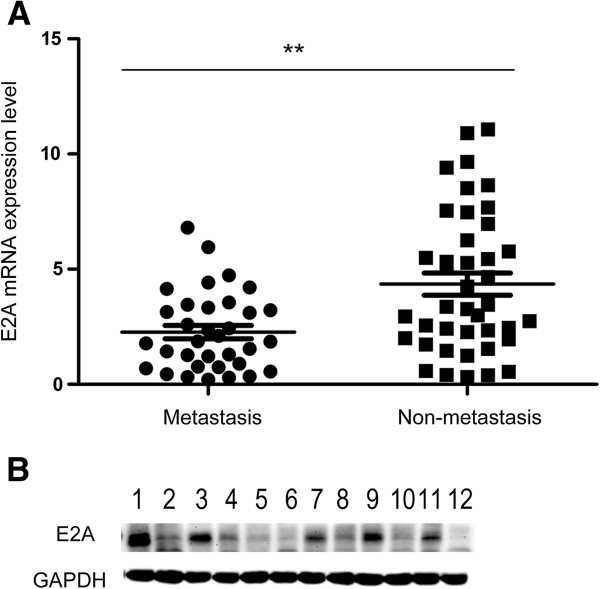
**Expression of E2A is decreased in metastatic CRCs. A**, Relative E2A mRNA expression in primary CRCs with or without metastasis. The levels of E2A mRNA were examined using qRT-PCR by the 2^-ΔCT^ method with GAPDH mRNA as internal control. **B**, E2A protein expression in primary CRCs with or without metastasis was detected with immunoblot using GAPDH protein as loading control. Protein samples of lane 1, 3, 5, 7, 9 and 11 were from tumors without metastasis, while lane 2, 4, 6, 8, 10 and 12 were from tumors with metastasis. Samples were selected and paired randomly. Data represents means ± SD from 3 independent experiments. **, *P* < 0.01.

To determine the role of E2A in CRC metastasis, we evaluated the mRNA expression level of E2A in 75 clinical specimens using qRT-PCR. Of the 75 cases, 43 patients were male and 32 were female with a median age of 56 years (range: 41–76 years); besides, 41 cases were metastasis negative and 34 were positive (including pathology confirmed lymph node and CT-scan detected distant metastases). As shown in Figure 1A, E2A mRNA expression was significantly decreased in tumors with metastases compared to those without (*p* < 0.01). We then made a correlation analysis to detect the relationship between E2A expression and clinicalpathological variables by classifying patients into E2A low or high group using the median E2A expression level as cutoff value. As shown in Table 1, expression of E2A was not related to gender (*p* = 0.653), age (*p* = 0.272), tumor histology (*p* = 0.410), or tumor site (*p* = 0.874), but inversely associated with advanced TNM stage (*p* = 0.010).

**Table 1:** Relationship between E2A expression level and clinicopathologic variables in CRC patients

**Variables**	**Subgroup**	**E2A expression**		***P *** **value**
		**Low**	**High**	
Gender	Male	26	17	0.653
	Female	21	11	
Age (years)	<60	22	15	0.272
	≧60	25	13	
Tumor histology	Tubular	39	21	0.410
	Mucinous	8	7	
Tumor site	Rectum and sigmoid	31	18	0.874
	Right colon	11	7	
	Left colon	5	3	
TNM stage	I	5	7	0.010
	II	16	13	
	III	18	7	
	IV	8	1	
Metastasis	Negative	21	20	0.024
	Positive	26	8	

To further confirm that E2A was also down-regulated at protein level in tumors with metastases, immunoblot was performed using 6 metastatic and 6 non-metastatic tumors chosen randomly from each group. As demonstrated in Figure 1B, metastatic tumors showed lower expression level of E2A protein. Taken together, lower E2A expression associates with positive metastatic status in CRCs.

### E2A suppressed CRC cells invasion and migration

**Figure 2:**
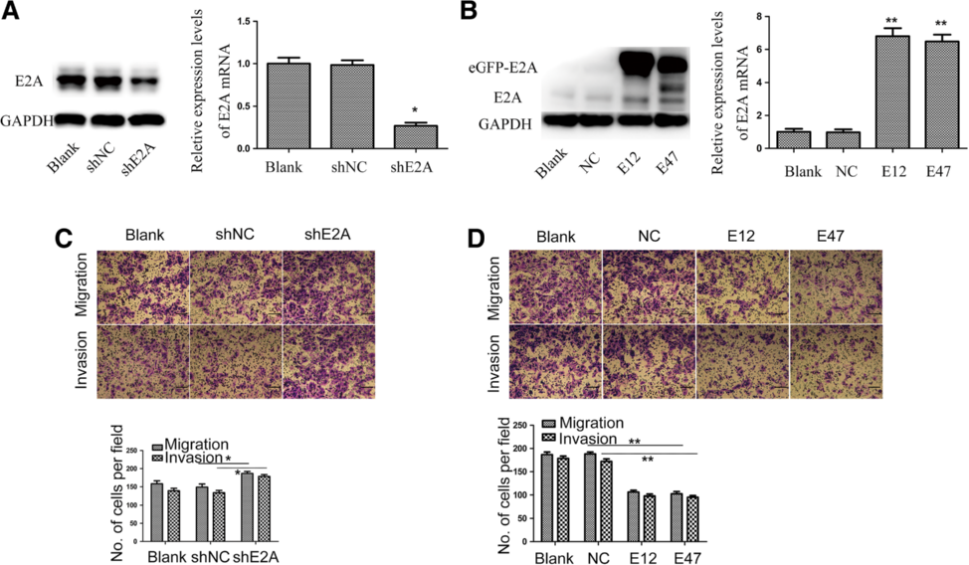
**E2A suppresses CRC cells invasion and migration. A**, Effect of shE2A on E2A expression. Left panel: Immunoblot showed E2A was reduced in shE2A treated SW480 cells compared to blank and shNC treated ones. GAPDH was used as loading control. Right panel: Relative E2A mRNA expression of shE2A-, shNC-treated and Blank SW480 cells. **B**, Transfection of plasmids pEZ-M29-E12 and pEZ-M29-E47 increased E2A mRNA and proteins expression. **C**, Downregualtion of E2A increased invasion and migration abilities of SW480 cells. **D**, Forced ectopic E12 or E47 expression suppressed invasion and migration abilities of SW480/shE2A cells. Representative photos of stained cells are shown with the original magnification of 100×. Scale bars: 100 μm Data represents the means ± SD from 3 independent experiments. *, *P* < 0.05; **, *P* < 0.01.

Next we wanted to know whether E2A was involved in regulation of CRC metastasis. To this end, SW480 cells were transfected with LV-shE2A to establish SW480/shE2A stable clones and LV-shNC was used as control. Transfection efficacy was verified by immunoblot and qRT-PCR (Figure 2A). Then we conducted cell invasion and migration assays. As shown in Figure 2B, downregulation of E2A increased the invasion and migration ability of SW480 cells by ~1.2 folds compared with the blank and shNC groups (*p* < 0.01). Given that E2A has two transcriptional variants E12 and E47, we went a step further by transiently transfecting SW480/shE2A cells with either pEZ-M29-E12 or pEZ-M29-E47 to ectopically express E12 or E47 to discover the isoform responsible for the suppression effect. The transfection efficacy was validated by immunoblot and qRT-PCR (Figure 2C). As demonstrated in Figure 2D, both E12 and E47 reduced invasion and migration of SW480/shE2A cells (*p* < 0.01); importantly, no significant differences in suppression effect between E12 and E47 were observed.

Then we used another colorectal cancer cell line, Caco-2, to investigate whether E2A exerted its function in a cell line specific manner. Similarly, we constructed two stable clones, Caco-2/shE2A (expressing shE2A) and Caco-2/shNC (expressing negative control) and as observed in SW480 cells, metastasis ability of Caco-2 cells increased upon shE2A transfection and was suppressed by E12 and E47 (Additional file 1: Figure S1A & B), suggesting the metastasis suppression effect of E2A was not cell line dependent. Hence, E2A was a metastasis suppressor gene in CRC.

### E2A inhibited the EMT program

**Figure 3:**
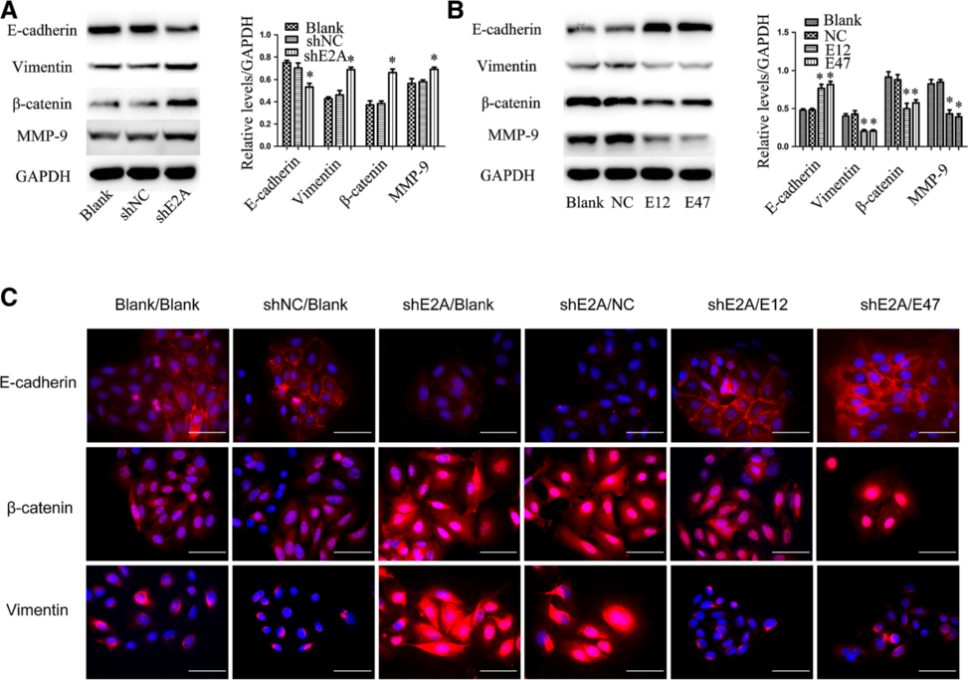
**E2A expression inhibits EMT in CRC cells. A**, Knockdown of E2A induced EMT and MMP-9 expression. Left panel: Immunoblot analysis showed epithelial marker E-cadherin was decreased and mesenchymal markers vimentin and β-catenin were increased in shE2A-expressing SW480 clone, indicating induction of EMT. MMP-9 was also increased upon E2A downregulation, which demonstrated enhanced invasion ability. GAPDH was used as loading control. Right panel: Densitometric analysis of left panel normalized to GAPDH. **B**, Expression of E12 and E47 suppressed EMT and MMP-9 expression. Left penal: Immunoblot analysis of E-cadherin, vimentin, β-catenin, MMP-9 and GAPDH expression with or without transfection of E12 or E47. Right penal: Densitometric analysis of left normalized to GAPDH. Data in the histograms is expressed as the means ± SD from 3 separate experiments. **C**, Immunofluorescence analysis using anti- E-cadherin, vimentin, β-catenin antibodies as indicated. Nuclei were counterstained with 4′, 6-diamidino-2-phenylindole (DAPI). Magnification: 400×; Scale: 50 μm. *, *P* < 0.05; **, *P* < 0.01.

In recent years, EMT has gained more attentions due to its importance in the acquisition metastatic potential during cancer progression [18-20]. Given the fact that E2A was decreased in metastatic CRCs and knockdown of E2A in CRC cells could promote invasion and migration, we wanted to know whether E2A could regulate EMT program in CRC cells. Indeed, expression of the epithelial marker E-cadherin was decreased and the mesenchymal markers vimentin and β-catenin were increased in SW480/shE2A cells (Figure 3A). In consistent with increased invasion ability, the expression of matrix metalloproteinases 9 (MMP-9) was elevated after downregulation of E2A (Figure 3A). Similarly, we transfected E12 and E47 plasmids separately into SW480/shE2A cells to identify which one was responsible for EMT regulation. As shown in Figure 3B, both E12 and E47 suppressed the transition induced by shE2A, with vimentin and β-catenin both reduced about fifty percent and E-cadherin enhanced by ~2 folds. Moreover, expression of these EMT makers didn’t show significant differences between E12- and E47-transfected SW480/shE2A cells (*p* > 0.05). Also, MMP-9 decreased after E12 and E47 transfection (Figure 3B).

To further demonstrate the role of E2A in EMT program regulation, we performed immunofluorescence to visualize these EMT markers in transfected SW480 cells. In coincidence with immunoblot results, immunofluorescence showed that E-cadherin was significantly decreased while vimentin and β-catenin were increased in SW480/shE2A cells compared with SW480 and SW480/shNC cells (Figure 3C). Furthermore, both E12 and E47 restored the expression of E-cadherin in SW480/shE2A cells while suppressed vimentin and β-catenin (Figure 3C). Thus, E2A may suppress invasion and migration through inhibiting EMT in CRC.

### YAP was a downstream target through which E2A suppressed metastasis

**Figure 4:**
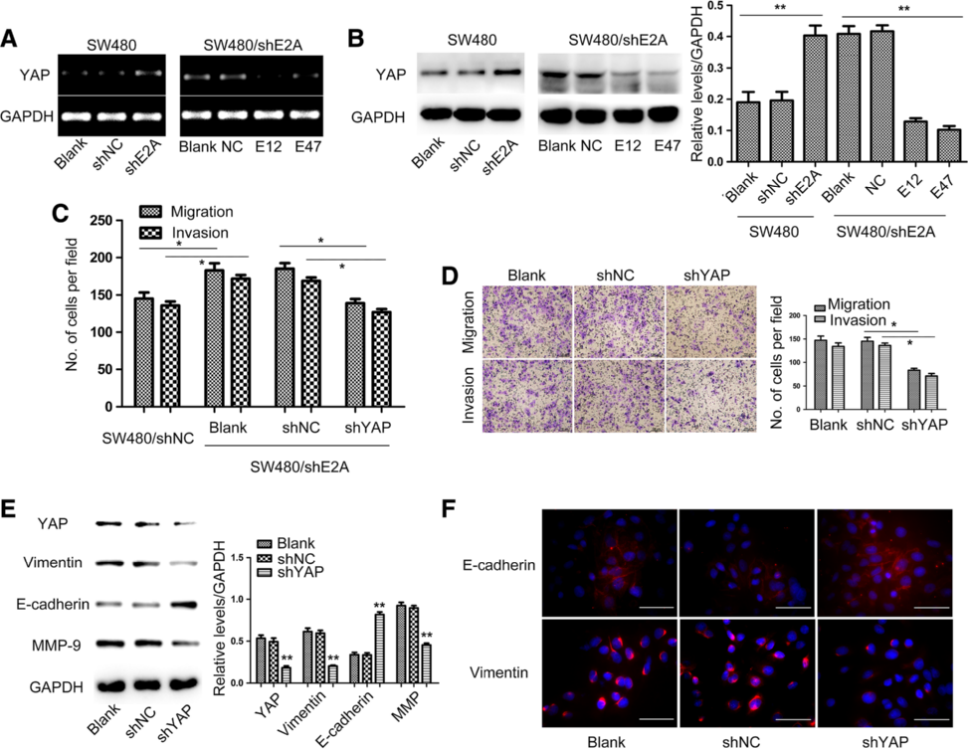
**Knockdown of YAP attenuates shE2A-induced invasion and migration. A**, Downregulation of E2A by shE2A resulted in increased YAP mRNA expression while transfection of plasmids encoding E12 or E47 decreased YAP mRNA in shE2A treated SW480 cells. **B**, E2A decreased YAP protein expression determined by immunoblot analysis. **C**, YAP knockdown by shYAP in SW480/shE2A cells suppressed the enhanced cell invasion and migration ability caused by shE2A to a similar level as observed in SW480/shNC cells. **D**, Downregulation of YAP by shYAP decreased the invasion and migration ability of SW480 cells. Migration/invasion ability of SW480 decreased after shYAP was transfected into SW480 cells. Representative photos of stained cells are shown with the original magnification of 100×. Scale bars: 100 μm. **E**, Immunoblot analysis of YAP, E-cadherin, vimentin, β-catenin and MMP-9 with GAPDH as loading control. Epithelial marker E-cadherin expression was elevated and mesenchymal marker vimentin and β-catenin expression was reduced after shYAP was transfected into SW480 cells. MMP-9 was decreased after shYAP transfection. Data in the histograms is expressed as the means ± SD from 3 separate experiments. **F**, Representative images of immunofluorescence analysis of EMT markers. E-cadherin was enhanced while vimentin and β-catenin were decreased after shYAP transfection. Magnification: 400×; Scale bars: 50 μm. *, *P* < 0.05; **, *P* < 0.01.

The findings above further led us to explore the potential molecules with which E2A interacted to regulate metastasis in CRC. As we described later, YAP (Yes-Associated Protein) was found to be one downstream target. We detected YAP mRNA expression in CRC tissues and found that YAP was inversely correlated with expression of E2A mRNA (*r* = 0.491, *p* < 0.01, Additional file 2: Figure S2), indicating YAP might be modulated by E2A in a suppressive manner. To find whether YAP was regulated by E2A, semi-qRT-RCR and immunoblot were performed to detect the expression of YAP mRNA and protein level after shE2A, E12 and E47 transfection. As shown in Figure 4A and 4B, YAP expression was increased both at mRNA and protein level in SW480/shE2A cells compared with control cells. Accordingly, both E12 and E47 plasmids attenuated shE2A-induced increase of YAP expression. Taken together, YAP was regulated by E2A.

Next, we asked whether the increased YAP in SW480/shE2A cells led to the enhanced cell aggressiveness. To this end, transient transfection of shYAP was performed in SW480/shE2A cells to down-regulate YAP (Additional file 3: Figure S3). As shown in Figure 4C, compared to cells transfected with negative control or blank cells, the invasion and migration ability of SW480/shE2A cells was significantly reduced by shYAP to the similar levels as observed in SW480/shNC cells. This finding suggested that the enhanced YAP by shE2A in SW480 cells was critical in the regulation of cell invasion and migration. Besides, downregulation of YAP impaired invasion and migration capacity of SW480 cells (Figure 4D). More importantly, MMP-9 expression was reduced to 50% of its normal level after shYAP transfection and changes of EMT markers, i.e. increased expression of E-cadherin and decreased vimentin, suggested a suppression of this program (Figure 4E). Immunoblot and immunofluorescence confirmed the expression alterations of E-cadherin and vimentin after shYAP transfection in SW480 cells (Figure 4E & F). Conclusively, YAP was a target through which E2A regulated EMT program to suppress invasion and migration in CRC cells.

## Discussion

Colorectal carcinogenesis is a multistep process mediated by complex cascades of molecular events governing genomic stability and cell proliferation [21]. Distant metastases, rather than the primary tumors from which these lesions arise, are responsible for >90% of carcinoma-associated mortality [22]. In the present study, we demonstrated the suppressive role of E2A in colorectal cancer cell invasion and migration; furthermore, YAP was demonstrated to be a downstream target of E2A in the metastasis of CRC cells.

E2A has been well described as a regulator of early B cell development, and it was dysregulated in lymphoma and breast cancer [13,23]. Decreased expression of E2A has been reported in metastatic pancreatic cancer cell lines [24]. In colorectal adenocarcinomas, ectopic expression of E47 results in proliferation inhibition [15]. The expression of E2A in CRCs is unknown and its role in CRC metastasis is also elusive. In this study, for the first time we investigated the association between E2A expression and CRC metastasis status and we found E2A was decreased in CRCs with metastases both at mRNA and protein levels, indicating its negative relation to CRC progression. *In vitro* invasion and migration assays also supported this: downregulation of E2A enhanced metastatic capacity of CRC cells while E12 and E47 could offset this effect. Taken together, E2A has a metastasis suppressive role in CRC. Additionally we found E2A may exert its action by regulating EMT. The EMT program plays an important role in tumor progression and metastasis [18,25]. Loss of epithelial traits and gain of mesenchymal features make epithelial tumor cells undergo morphological changes and acquire enhanced metastatic abilities. In our study, we found E2A downregulation inhibited the expression of epithelial marker E-cadherin and increased mesenchymal markers vimentin and β-catenin in SW480 cells, indicating EMT suppression by E2A. Considering that E-cadherin was regulated by multiple signal pathways [26], we speculate enhanced β-catenin expression was the main reason for decreased E-cadherin. However, the definite role of E2A in EMT regulation remains further study.

In further investigating the mechanism of action of E2A, we found YAP was regulated as a downstream target. The YAP gene is located on chromosome 11q22, a region which has been described in previous studies to be amplified in several types of cancers [27,28]. As one of the highly conserved components in mammals, YAP has been proved to be a nuclear effector of the Hippo pathway and was initially identified by mosaic screens in Drosophila melanogaster as a vital growth regulator of cell proliferation and apoptosis [29-31]. YAP is also a transcriptional modulator which has been implicated in stem cell differentiation, control of organ size, and tumor growth [32,33]. colonic adenocarcinoma tissues show up-regulated YAP expression compared with normal colon tissues, and inducible transgenic expression of a stabilized YAP mutant (S127A) in mice induced colonic adenomas [34,35]. Indeed, Wang et al. found that YAP was a prognostic marker of CRC and down-regulation of YAP reduced the metastatic ability of CRC cells [36]. In our study, we found YAP was inversely associated with E2A in CRC tissues. This further led us to discover that YAP was a downstream target of E2A as its expression was increased upon shE2A transfection while E12 and E47 transfection could reduce it to normal level. Moreover, β-catenin, which was regulated by E2A, could enhance YAP expression by directly binding to YAP gene in CRC cells [37]. In the present study, we found YAP exerted its function of enhancing metastasis by inducing EMT in CRC cells, which was in consistent with the work of Wang et al. [36]. Importantly, knockdown of YAP in shE2A treated SW480 cells could abolish the elevated cell invasion and migration caused by shE2A. This finding suggested the role of YAP in the E2A regulated inhibition of cell invasion and migration. Hence, YAP plays as a downstream in mediating E2A’s function as a tumor suppressive gene in CRC.

## Conclusion

The findings of our study suggest that E2A expression is associated with CRC metastasis. By targeting YAP, E2A inhibits EMT program and suppresses invasion and migration in CRC cells. Although E2A’s function in cancer has not been fully understood, our findings provide new molecular target and mechanism of action of E2A in CRC metastasis. Therefore, E2A has the potential value to be developed as a new target for CRC prevention and therapy.

## Competing interests

The authors declare that they have no competing interests.

## Authors’ contributions

HCZ, AH and PL carried out the experimental studies, drafted the graphs, performed the statistical analysis and wrote the paper. BF and YJQ provided experimental technical supports. ZHM, ZGZ, XHC and MHZ contributed to the study design and manuscript revision, and provided funding for this study. All authors read and approved the final manuscript.

## Supplementary Material

Additional file 1: Figure S1 E2A suppresses Caco-2 invasion and migration. A, shE2A expression suppresses invasion and migration of Caco-2 cells. B, E12 or E47 expression decreased cell invasion and migration of Caco-2/shE2A cells. Data represents the means ± SD from 3 independent experiments. Representative photos of stained cells are shown with the original magnification of 100×. Scale bars: 100 μm. *, *P* < 0.05. Click here for file

Additional file 2: Figure S2 Significant inverse correlation between YAP mRNA expression and E2A mRNA expression (Pearson’s correlation R = -0.491, P < 0.01). Click here for file

Additional file 3: Figure S3 shYAP decreased the expression of YAP protein in SW480/shE2A cells. Upper penal: Immunoblot analysis using anti-YAP antibodies as indicated with or without shYAP expression. GAPDH was used as loading control. Lower panel: Densitometric analysis of upper panel normalized to GAPDH. Click here for file
