# Supplementary figures and images for: E2A suppresses invasion and migration by targeting YAP in colorectal cancer cells
Source: J Transl Med. 2013 Dec 26;11:317. doi: 10.1186/1479-5876-11-317 (PMC3879192; doi:10.1186/1479-5876-11-317)

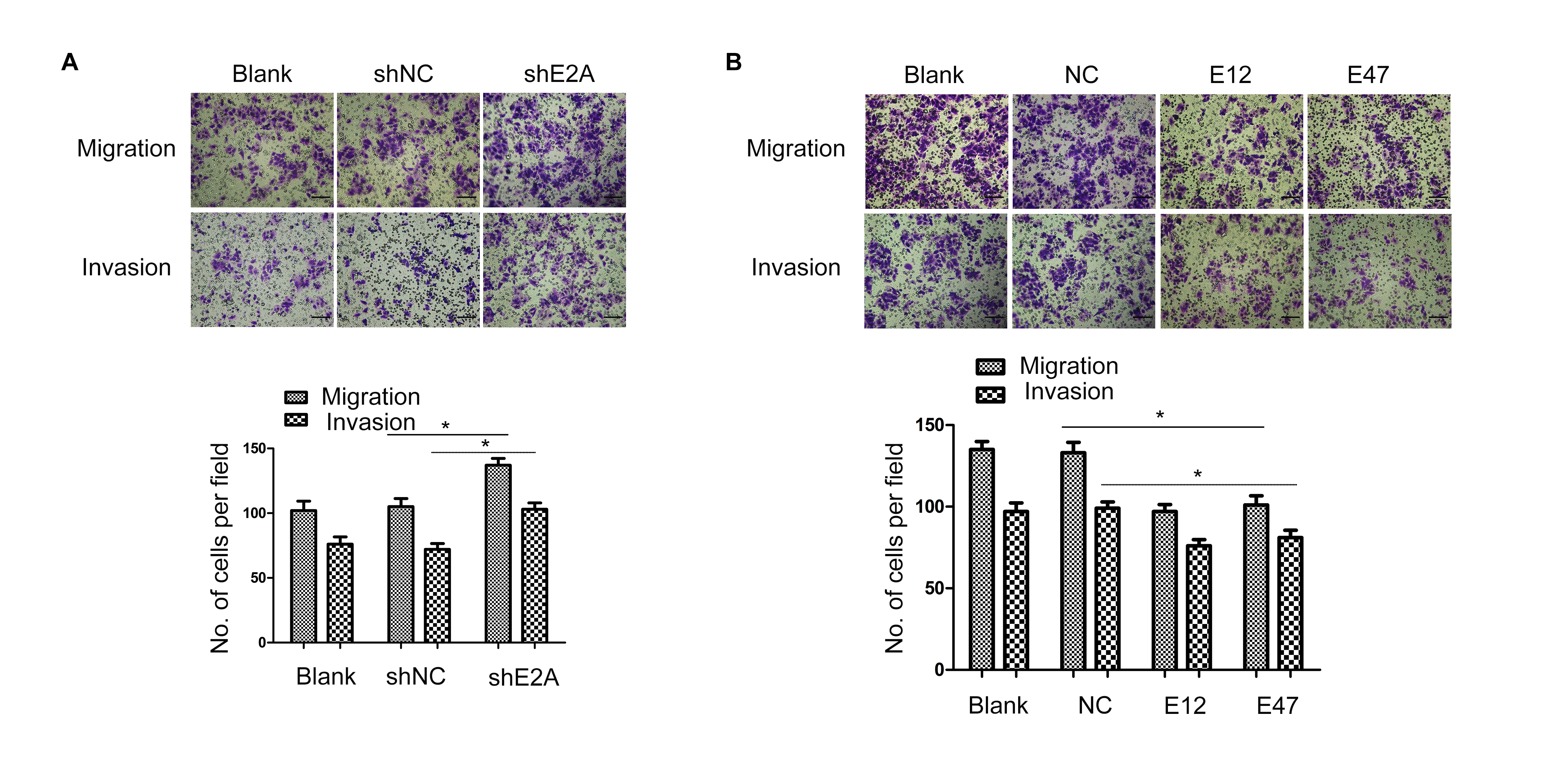

Supplement: Additional file 1: Figure S1 — E2A suppresses Caco-2 invasion and migration. A, shE2A expression suppresses invasion and migration of Caco-2 cells. B, E12 or E47 expression decreased cell invasion and migration of Caco-2/shE2A cells. Data represents the means ± SD from 3 independent experiments. Representative photos of stained cells are shown with the original magnification of 100×. Scale bars: 100 μm. *, P < 0.05. [file 1479-5876-11-317-S1.tiff]

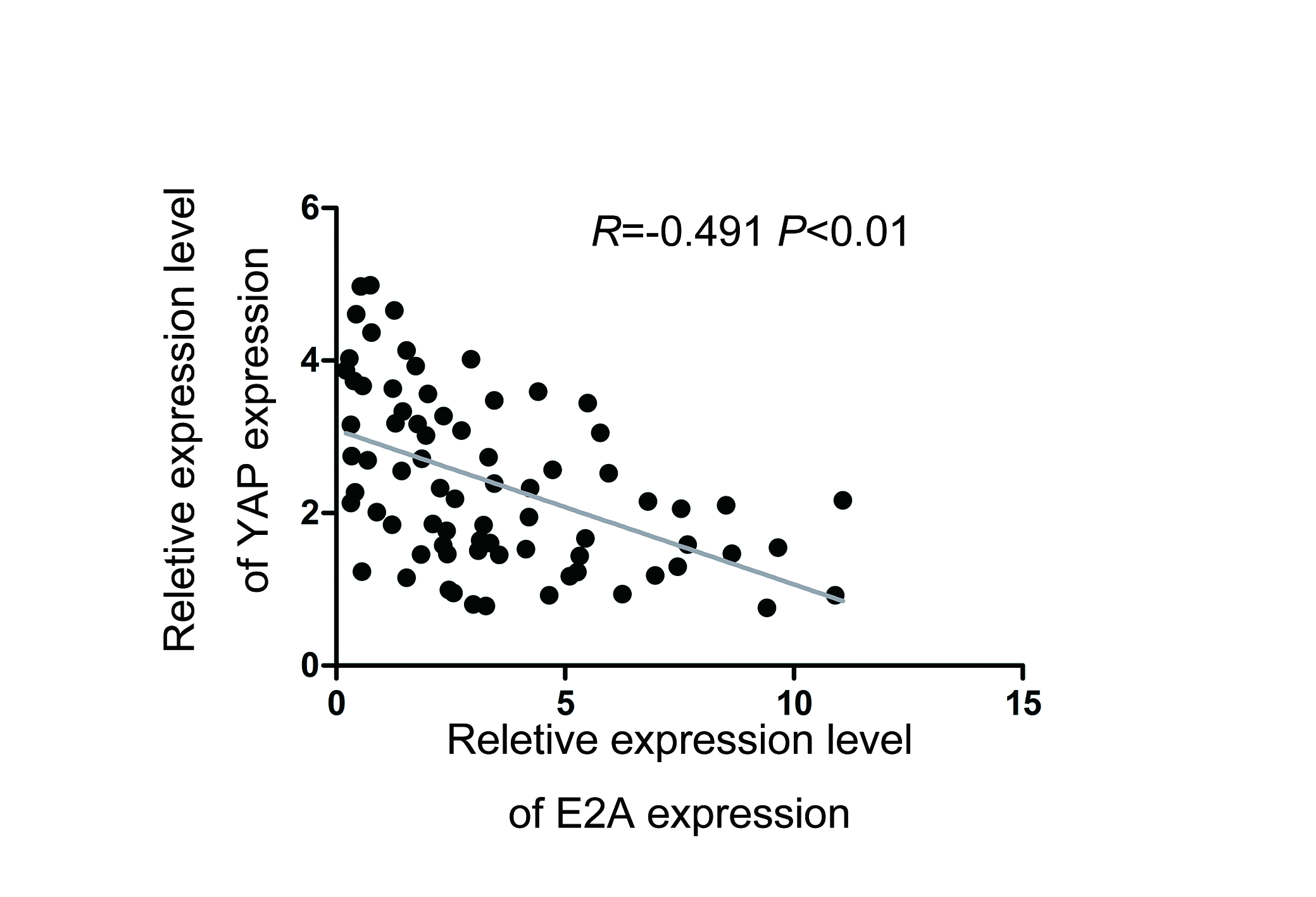

Supplement: Additional file 2: Figure S2 — Significant inverse correlation between YAP mRNA expression and E2A mRNA expression (Pearson’s correlation R = -0.491, P < 0.01). [file 1479-5876-11-317-S2.tiff]

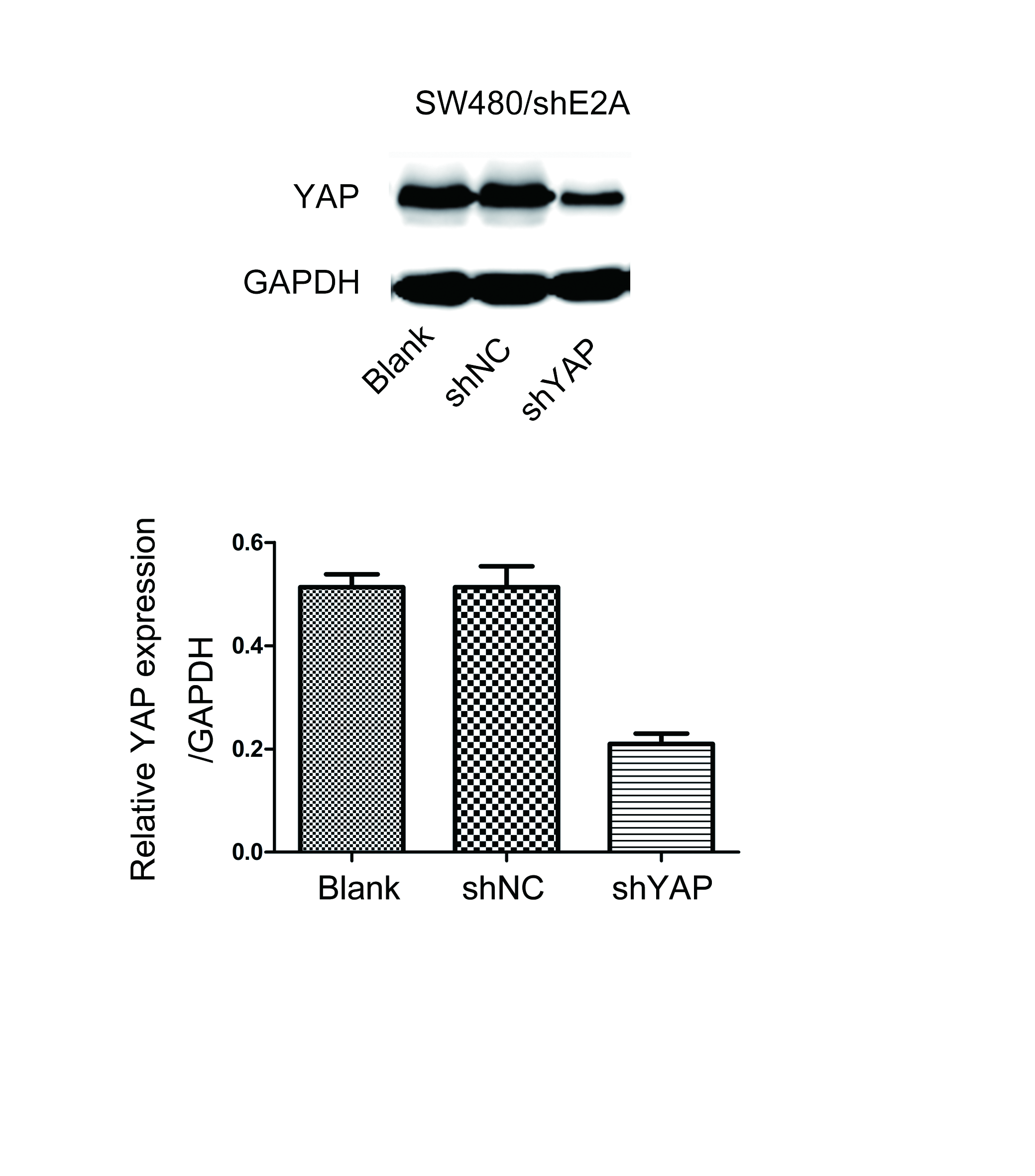

Supplement: Additional file 3: Figure S3 — shYAP decreased the expression of YAP protein in SW480/shE2A cells. Upper penal: Immunoblot analysis using anti-YAP antibodies as indicated with or without shYAP expression. GAPDH was used as loading control. Lower panel: Densitometric analysis of upper panel normalized to GAPDH. [file 1479-5876-11-317-S3.tiff]
